# Drone Detection and Tracking Using RF Identification Signals

**DOI:** 10.3390/s23177650

**Published:** 2023-09-04

**Authors:** Driss Aouladhadj, Ettien Kpre, Virginie Deniau, Aymane Kharchouf, Christophe Gransart, Christophe Gaquière

**Affiliations:** 1 COSYS-LEOST, Université Gustave Eiffel, 20 Rue Élisée Reclus, 59650 Villeneuve-d’Ascq, France; virginie.deniau@univ-eiffel.fr (V.D.); christophe.gransart@univ-eiffel.fr (C.G.); 2 MC2 Technologies, 1 Rue Héraclès, 59493 Villeneuve-d’Ascq, France; ekpre@mc2-technologies.com (E.K.); akharchouf@mc2-technologies.com (A.K.); cgaquiere@mc2-technologies.com (C.G.)

**Keywords:** drone, UAV, C-UAS, RF signal, Drone ID, detection system, tracking system, drone position, distance estimation, reaction time

## Abstract

The market for unmanned aerial systems (UASs) has grown considerably worldwide, but their ability to transmit sensitive information poses a threat to public safety. To counter these threats, authorities, and anti-drone organizations are ensuring that UASs comply with regulations, focusing on strategies to mitigate the risks associated with malicious drones. This study presents a technique for detecting drone models using identification (ID) tags in radio frequency (RF) signals, enabling the extraction of real-time telemetry data through the decoding of Drone ID packets. The system, implemented with a development board, facilitates efficient drone tracking. The results of a measurement campaign performance evaluation include maximum detection distances of 1.3 km for the Mavic Air, 1.5 km for the Mavic 3, and 3.7 km for the Mavic 2 Pro. The system accurately estimates a drone’s 2D position, altitude, and speed in real time. Thanks to the decoding of telemetry packets, the system demonstrates promising accuracy, with worst-case distances between estimated and actual drone positions of 35 m for the Mavic 2 Pro, 17 m for the Mavic Air, and 15 m for the Mavic 3. In addition, there is a relative error of 14% for altitude measurements and 7% for speed measurements. The reaction times calculated to secure a vulnerable site within a 200 m radius are 1.83 min (Mavic Air), 1.03 min (Mavic 3), and 2.92 min (Mavic 2 Pro). This system is proving effective in addressing emerging concerns about drone-related threats, helping to improve public safety and security.

## 1. Introduction

Unmanned aerial vehicles (UAVs) commonly known as drones, are becoming omnipresent in various industries due to their versatility and sophistication. By integrating advanced technologies, such as modular software architecture, and a multitude of sensors (Global Positioning System (GPS), light detection and ranging (LiDAR), radio detection and ranging (RaDaR), and visual sensors), drones can perform a wide range of tasks, from surveillance and videography [1] to agriculture monitoring [2], delivery services [3], and aiding in health emergencies [4,5]. These flying machines offer numerous benefits, including stability, ease of piloting, and autonomous flight [6] with pre-programmed flight data. Another advantage of drones is their ability to fly in large numbers and communicate efficiently [7], taking advantage of swarm intelligence techniques [8] often used in optimization problems. However, the widespread use of drones has also led to their exploitation in malicious activities [9], including drug trafficking [10], smuggling, and bombing [11]. These activities pose a significant threat to public safety. Thus, it is required to detect the presence of unauthorized drones to fight against these malicious activities.

**Figure 1:**
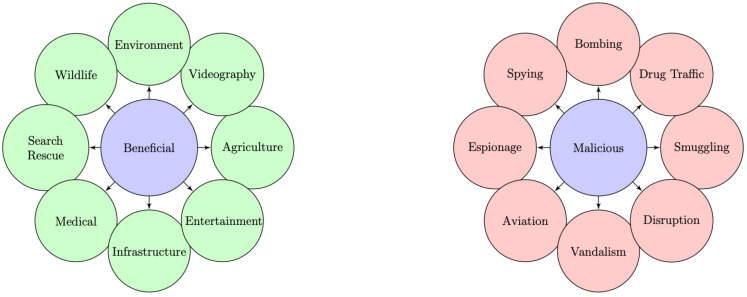
Summary of beneficial and malicious uses of drones.

Figure 1 illustrates the multifaceted applications of drones, highlighting both their beneficial and malicious uses. To distinguish between drones used for legitimate or malicious activities, accurate drone detection and tracking systems are required. These systems can enable countermeasures to be activated in good time. To address these challenges, counter-unmanned aerial systems (C-UASs) [9,12,13,14,15,16,17,18] have to be developed. Effective detection, tracking, and recognition solutions [19,20,21] are essential if any suspicious drone activity is to be neutralized.

This article focuses on drone detection through RF spectrum monitoring [22]. It presents a comprehensive and advanced approach to detect and decode Drone ID signals [23]. To assess the effectiveness of the proposed system, a measurement campaign was carried out over a larger area, involving three drones: DJI Mavic Air, DJI Mavic 2 Pro, and DJI Mavic 3, equipped with RF Drone ID signals. These drone models offer insights into the types of drones that may see widespread adoption in the future among both professional and amateur users.

## 2. Background and Related Work

Different technologies can be exploited for detecting and tracking drones. UAV detection methods, based on imagery and radar sensors, necessarily work in line of sight (LoS) conditions. In urban environments, drones can navigate without being detected by these methods because they can be masked by buildings. In addition, in vast open areas, these methods can have high detection distances but may lead to false detection due to confusion with other flying entities, such as birds. Acoustic technologies, meanwhile, suffer from limited detection range and ambient noise susceptibility. A more exhaustive overview of drone detection methodologies can be found in Table 1.

The passive RF detection method relies on spectral surveillance to identify the communications between the drone and its remote control (RC) within the electromagnetic spectrum. These methods do not require LoS. However, passive RF detection faces challenges when the signals emitted by the drone coexist with numerous other signals, such as Wi-Fi or Bluetooth, which share the same frequency band. This presents a challenge in attributing each signal to its respective emitter, especially in complex urban environments. Consequently, it necessitates the collection of RF communication data from each RF source and the construction of a comprehensive database encompassing various scenarios and diverse environments to enhance detection capabilities for more general cases. To address these challenges, recent studies have made notable contributions. In 2019, Al-Sa’d et al. [34] introduced an open-source drone database for RF-based detection and identification, demonstrating the effectiveness of deep neural networks in achieving high accuracy rates. In 2020, Feng et al. [35] proposed an efficient two-step method for detecting drone hijacking using a combination of genetic algorithm-extreme gradient boosting (GA-XGBoost) with GPS and inertial measurement unit (IMU) data, achieving high prediction correctness and time savings. In 2021, the study conducted by Basak et al. [31] introduced a two-stage approach. The detection stage employed goodness-of-fit (GoF) sensing, while the classification stage utilized the deep recurrent neural network (DRNN) framework. They developed a customized you only look once (YOLO)-lite framework from scratch to achieve integrated drone RF signal detection, spectrum localization, and classification. The performance of both techniques was evaluated using a newly created drone dataset, demonstrating favorable results in terms of detection and classification. However, it is important to note that since the classification was conducted in a supervised manner, the performance may vary when encountering unknown or newer drone signals, as highlighted in the limitations. In 2022, Medaiyese et al. [36] employed wavelet transform analytics and machine learning for RF-based UAV detection, achieving 98.9% accuracy with an image-based signature and a pre-trained convolutional neural network (CNN)-based model. Kılıç et al. [37] also focused on drone classification based on RF signals, achieving high accuracy rates using spectral-based audio features and a support vector machine (SVM)-based machine learning algorithm. In the same year, Sazdic-Jotic et al. [38] presented a method for single and multiple drone detection and identification using RF-based deep learning algorithms, achieving high accuracy in both scenarios.

Previous methods used for drone detection and classification exhibit suboptimal performance. First, to integrate new drones into the full database, it is necessary to study and record all potential communication scenarios and take into account different channel and multipath models [32,34,39]. However, this approach can introduce limitations in terms of flexibility and operational efficiency. Moreover, they rely heavily on AI algorithms that operate on large data sets, resulting in a procedure that requires significant training and consumes considerable memory resources. Furthermore, the previous techniques concentrate only on the processing of the raw modulated signals without the possibility of analyzing the data encoded in the communication protocol. Therefore, they cannot extract crucial information relevant to the defense industry, such as the device manufacturer, the location of the drone, and the purpose and mission of the flight. In this perspective of analyzing the data link layer and the network layer, some recent research has contributed to the development of this idea. Christof [40] reverse-engineered the Wi-Fi protocol of DJI drones. Using deductive and bit-perfect analysis, he was able to determine the structure of the protocol and extract information using specially developed open-source software. This information can be crucial for detecting and locating drones in real time. In addition, Bender [41] and the Department 13 article [42] demonstrated that DJI Drone IDs are not encrypted. This discovery is essential for drone detection, as it allows DJI drones to be spotted and identified using dedicated software-defined radio (SDR). The author proposed a real-time DJI OcuSync Drone ID detection system, using low-cost SDRs with robust packet analysis. This detection system was found to be much cheaper than DJI AeroScope [43], which is priced between USD 20,000 and USD 40,000. In addition, the detection system not only allows model identification but also the retrieval of serial numbers and telemetry information from DJI drones. However, this system’s detection range is currently limited to 1.2 km.

The originality of the proposed solution and the work presented in this article is summarized as follows:

- Addressing a method that allows integrating drone detection, classification, and localization solutions into a single module.
- Proposing a complete system integrating both hardware components and software tools and capable of detecting some recent drones.
- Carrying out a long-distance measurement campaign to assess detection performance in terms of distance and altitude.
- Providing real-time estimation of drone localization parameters, including position, velocity, and altitude. The Haversine formulas are used to estimate the remaining distance between the system and the detected drone.
- Providing an estimated remaining reaction time in the context of securing an area with a specified radius.

This research paper delves into drone detection and tracking through RF spectrum monitoring, offering an approach to decode drone identification signals. The proposed system integrates both hardware components and software tools to accomplish detection and tracking tasks. A measurement campaign involving three drones has been executed to evaluate the system’s efficacy in terms of range detection, estimating the altitude and velocity of each drone, their trajectory, and finally, their remaining time to penetrate a secured zone protected by this system.

The rest of the paper is organized as follows: We present the RF communication protocols commonly used by drones in Section 3, including details about Drone ID packets. The general methodology underlying our implemented drone detection and tracking algorithms is presented in Section 4. We then describe the hardware components and software tools used for monitoring, signal processing, and analysis in Section 5 and Section 6, respectively. For the experimental setup involving three drones, we elaborate on the configuration in Section 7. The methodology for conducting measurements and evaluating the system’s performance is detailed in Section 8. In Section 9, we provide a comprehensive analysis and interpretation of the obtained results. Finally, we conclude the paper in Section 10, highlighting the strengths and limitations of our detection solution and providing suggestions for future improvements.

## 3. Drone Communication Protocols

Most UASs utilize RF transmissions for communication between the UAV and its associated RC [44]. This bi-directional communication involves both uplink and downlink signals, allowing for seamless information exchange. It enables the transfer of precise control commands, encompassing throttle, pitch, yaw, and roll, to ensure accurate maneuvering of the UAV. Furthermore, it facilitates the exchange of crucial information with the pilot, including UAV position, remaining flight time, distance to target and pilot, payload specifics, speed, altitude, and video imagery. Additionally, it supports the transmission of flight missions, acknowledgments, and protocol-dependent data, expanding the scope of control commands beyond the drone’s speed coordinates.

The drone transmission system typically operates in the industrial, scientific, and medical (ISM) bands, and the frequency choice depends on the geographical location of the drone. For example, in France, the 2.4 GHz band offers a wide coverage area but a slower data transmission speed, while the 5.8 GHz band provides a faster data speed but a more limited coverage area.

Several communication protocols can be used to establish the RF link between the drone and its RC, including Wi-Fi, enhanced Wi-Fi, Lightbridge, and OcuSync. The drone’s range, video transmission quality, latency, available control frequencies, and other related parameters all depend heavily on the communication protocol employed.

### 3.1. Wi-Fi and Enhanced Wi-Fi Communication Protocols

The use of standard Wi-Fi in drones offers an efficient and cost-effective control method for many manufacturers. This standard utilizes the conventional IEEE Wi-Fi 802.11 network to connect the drone and a control device. The drone creates a private Wi-Fi network, which users can access by providing a password. This Wi-Fi connection lets users control the possibility of controlling the drone using a dedicated RC, a cell phone, or a tablet.

Some drones on the market, such as DJI’s Spark, Mavic Air, DJI Mini, and Mini SE models, employ Wi-Fi technology for connectivity. They support two frequency bands, 2.4 GHz and 5.8 GHz, and the system intelligently switches between them for optimal control. DJI offers two Wi-Fi connectivity options for these drones: standard Wi-Fi for the Spark model and enhanced Wi-Fi for the Mavic Air, Mini, and Mini SE drones. Standard Wi-Fi connectivity provides a transmission range of up to 500 m for the Spark, while enhanced Wi-Fi connectivity enables the Mavic Air, Mini, and Mini SE to achieve a transmission range of up to 2000 m. Both Wi-Fi systems support 720 p video transmission, providing users with a clear view of the drone’s camera for surveillance, shooting, and other applications.

Moreover, drone manufacturers have also developed proprietary or enhanced Wi-Fi protocols to optimize performance and enhance the user experience. These protocols offer features such as extended range, reduced latency, and greater resistance to interference, ensuring more robust connectivity for drone operations [45].

### 3.2. Lightbridge Communication Protocol

In response to the limitations of Wi-Fi for professional drone applications, DJI made a strategic shift towards developing the Lightbridge communication protocol. This move was driven by the need for enhanced performance, reliability, and range in professional and enterprise-level drone operations. Wi-Fi, while suitable for consumer-grade drones, often faces challenges in terms of signal stability, latency, and limited range. By introducing Lightbridge, DJI aimed to address these limitations and provide a robust communication solution for their professional drone lineup.

Lightbridge drones utilize a dual-band transmission system, operating on both the 2.4 GHz and 5.8 GHz frequency bands. This dual-band capability allows for improved signal resilience and flexibility, as the drones can intelligently switch between the two frequency bands based on the environmental conditions and interference levels. The transmission system ensures reliable and low-latency video transmission, providing pilots with a clear and real-time view from the drone’s camera.

The Lightbridge communication protocol offers two main versions: Lightbridge HD and Lightbridge HD 2 [46]. These versions are implemented in various DJI drone models, including the Phantom 4 Pro, Phantom 4 Advanced, Inspire 2, Matrice 200 Series, and Matrice 600 Pro. Lightbridge-equipped drones are capable of transmitting signals over an extended range, reaching up to 3.6 km in countries subject to CE regulations.

Lightbridge drones leverage advanced features to deliver high-quality video transmission and responsive control for professional aerial photography, cinematography, and industrial applications. With this protocol, DJI has significantly improved the communication capabilities of their drones compared to the Wi-Fi and the enhanced Wi-Fi protocols, offering professionals a reliable and efficient tool for their work [45].

### 3.3. OcuSync Communication Protocol

The OcuSync protocol, developed by DJI, is widely employed in the latest consumer and enterprise models of their drones [47]. It offers an extended transmission range compared to both the Wi-Fi and Lightbridge protocols.

OcuSync utilizes a multi-band, multi-service, and multi-channel approach to ensure optimal stability and data throughput. Its multi-service system enables simultaneous transmission of video, control, and telemetry signals. With the implementation of orthogonal frequency division multiplexing (OFDM), the video signal can withstand interference or attenuation, delivering satisfactory performance even over long distances. Furthermore, the protocol incorporates automatic channel switching, seamlessly transitioning to less congested channels when interference surpasses a certain threshold. This ensures uninterrupted video transmission, while the control and telemetry signals utilize frequency hopping spread spectrum (FHSS) modulation. By employing FHSS, packets are sent using random frequencies that regularly change, enhancing resistance to packet loss [40].

DJI has consistently enhanced its OcuSync transmission system over time [48]. It was initially introduced in the Mavic Pro, followed by the improved OcuSync 2.0 [46] in the Mavic 2 Pro/Zoom and Mini 2 drones. The latest advancements include OcuSync 3.0, OcuSync 3.0+, and OcuSync 3.0 Enterprise, featured in drones such as the Mavic 3, Mini 3 Pro, M30 series, and M300 RTK. These drones benefit from OcuSync’s advanced capabilities, providing users with reliable, high-quality transmission for a wide range of applications [45].

### 3.4. Drone Specific Packets

#### 3.4.1. RDID Packet

Remote drone identification (RDID) is now an essential global regulatory framework implemented in regions such as the USA, Europe, France, and Japan, following international standards such as American Society for Testing and Materials (ASTM) and Aerospace and Defence Industries Association of Europe (ASD-STAN). The Federal Aviation Administration (FAA) introduced the RDID rule in April 2021, making real-time identification and tracking of drones, operators, and ground control stations mandatory. Drone manufacturers must comply with the RDID standard by September 2022, and operators have until September 2023 [49].

By broadcasting identification codes, position data, and emergency status, RDID enables effective detection and tracking of drones. This improves safety, security, and regulatory compliance. Drones can achieve RDID compliance through network-based approaches using persistent internet connections or broadcast-based approaches using Wi-Fi or Bluetooth technologies. Specific areas, called FAA-recognized identification areas (FRIA), allow operation without an RDID module. International regulations, such as those of Europe, France, and Japan, address RDID requirements and impose the dissemination of unique identification serial numbers, locations, and operator information. The guidelines provided by ASTM and ASD-STAN focus on the dissemination of drone identity and global navigation satellite systems (GNSS) location using Bluetooth and Wi-Fi technologies [50]. An example of implementing RDID can be found in the repository available in [51].

#### 3.4.2. DJI Drone ID Packets

DJI has launched the transmission of a private drone identifier for several reasons. The use of a localized, unconnected identifier associated with a specific drone enables seamless integration of public safety, security, and drone operator liability while guaranteeing operator privacy and security. To achieve this, DJI uses two exclusive communication protocols, enhanced Wi-Fi and OcuSync, to transmit the DJI Drone ID signal.

On the one hand, the DJI Drone ID enhanced Wi-Fi signal occupies a bandwidth of 5 MHz and uses FHSS modulation on the 2.4 GHz and 5.8 GHz bands. DJI incorporates this identification packet into IEEE 802.11 Wi-Fi beacon management frames, which are designed to announce the presence of vehicles.

On the other hand, the DJI Drone ID OcuSync is transmitted by the drone using the same hardware as its communication. It occupies a bandwidth of 10 MHz and utilizes FHSS modulation on the 2.4 GHz and 5.8 GHz bands. Notably, even if a user forces the DJI OcuSync communication downlink to operate on 2.4 GHz or 5.8 GHz using the DJI smartphone app, the DJI Drone’s identification signals continue to be broadcast out-of-band via the communication link [41].

## 4. Drone Detection and Tracking Methodology

As mentioned previously, the communication protocol varies from one drone to another. This article focuses on drones that use the Wi-Fi standard or enhanced Wi-Fi for communication, as well as drones equipped with Wi-Fi Remote ID or enhanced Wi-Fi DJI Drone ID signals.

**Figure 2:**
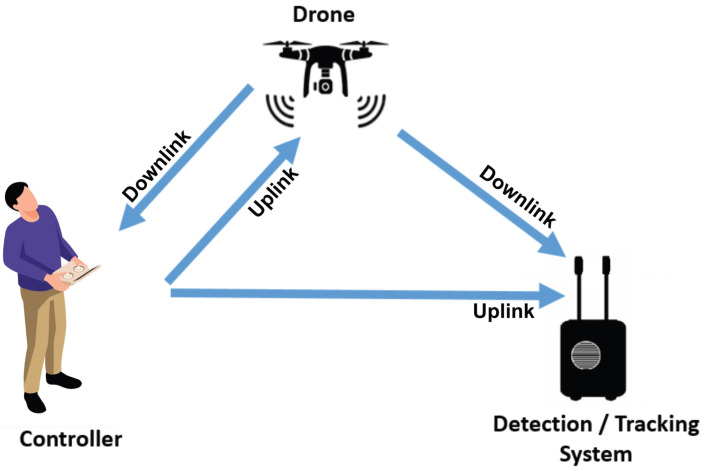
Detection and tracking scenario: downlink (video and telemetry)/uplink (control).

Figure 2 illustrates a scenario of malicious use of a drone, with a remote pilot controlling the drone while hovering or flying near a vulnerable site. To ensure effective protection, the detection system has to be in the area to protect. This system has to be able to detect the uplink and downlink signals and relay information about the drone’s presence and location to a central server. The range of detection depends on factors such as the RF amplification chain, hardware components, and software processes involved in the system.

Thus, regardless of whether a drone complies with regulations or not, the system should detect all drones operating within its vicinity. Taking into account these different drones, we break down the problem into three detection cases:

- Drones that communicate using the Wi-Fi standard protocol within the ISM band.
- Drones that transmit a Wi-Fi RDID beacon on channel 6 (at 2437 MHz) within the 2.4 GHz Wi-Fi band.
- DJI drones that transmit the enhanced Wi-Fi DJI Drone ID signal. The specific channel used by these drones is pseudo-random and can be within either the 2.4 GHz or 5.8 GHz ISM band.

This article does not cover DJI drones emitting the OcuSync DJI Drone ID [41], nor drones using communication protocols for which decoding methods are currently undergoing reverse-engineering processes.

## 5. Detection and Tracking Hardware System

In this section, we present the hardware equipment used to monitor the frequency bands and detect drones with their ID signals. The system comprises various components designed specifically for this purpose.

### 5.1. Jetson Nano Development Kit

In this study, the Jetson Nano developer kit was selected to constitute the processing unit of the detection system. The Jetson Nano platform hosts all the algorithms and software components necessary for the acquisition, analysis, and decoding of signals. The performance of the Jetson Nano kit may not be essential for the developments described in this article. However, the selection of the Jetson was made with the expectation of the potential utilization of AI algorithms in the future to detect and track drones with unknown protocols.

### 5.2. Wi-Fi Receiver for RF Monitoring

With the aim of drone detection through Wi-Fi standard and enhanced Wi-Fi protocols, specific receivers are used to detect these signals, as the Jetson card only serves for data processing and not for data acquisition. This prototype features two distinct RF Wi-Fi receivers. The first one is specifically designed to detect frequency hopping DJI Drone ID and standard Wi-Fi communication. The other one is set to detect the RDID fixed at 2437 MHz.

**Figure 3:**
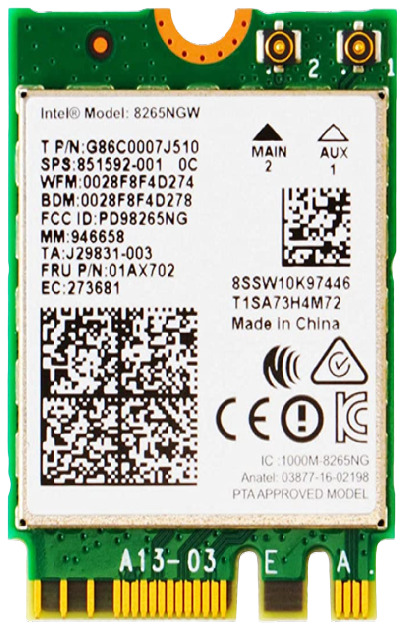
Intel wireless chipset.

**Figure 4:**
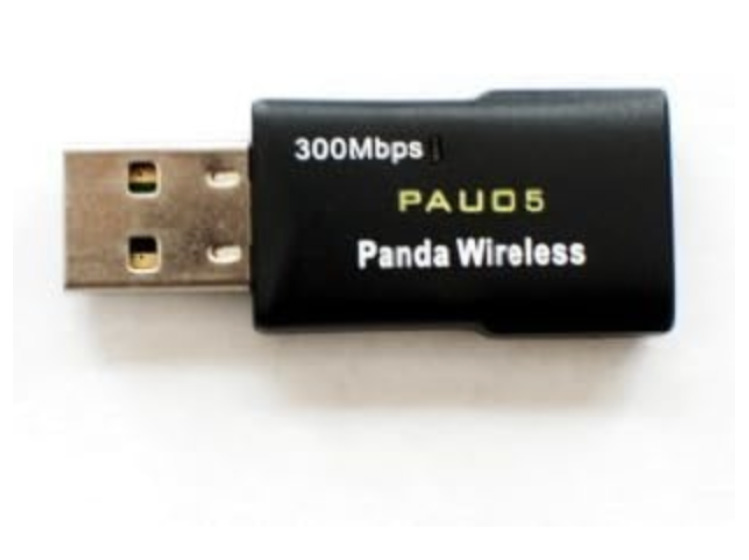
Panda wireless chipset.

As shown in Figure 3 and Figure 4, both the Intel 8265 [52] and the Panda [53] Wi-Fi boards are capable of detecting Wi-Fi packets. On the one hand, the Intel card has an advantage over other Wi-Fi chips because it can scan a wide range of Wi-Fi channels, from 1 to 177, covering frequencies of 2.4 GHz and 5.8 GHz, with instantaneous bandwidths of 5 MHz, 10 MHz, 20 MHz, and 40 MHz.

On the other hand, the Panda Wi-Fi card offers a coaxial SMA RF connector for connecting an optimized Rx chain, and supports only 2.4 GHz frequencies, making it ideal for long-range wireless network deployments. Moreover, it offers a throughput of up to 300 MB per second. Both Wi-Fi cards can monitor and intercept Wi-Fi packets, enabling access to the drone’s data. Wireless card performance has a significant impact on range and accuracy. Choosing the right Wi-Fi card is, therefore, essential to build an effective drone detection and tracking system.

### 5.3. Radio Receiver Architecture

**Figure 5:**
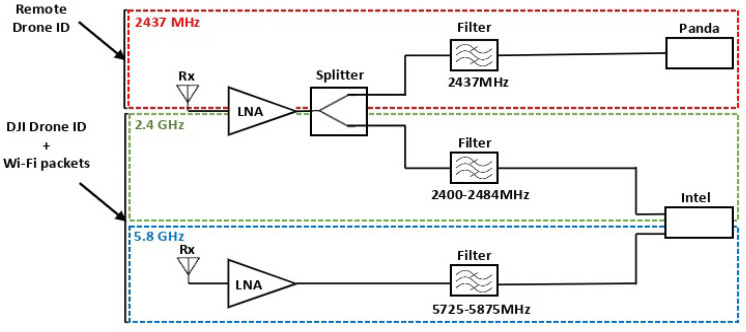
Dual-band RF receiver architecture.

Wi-Fi signals can be strongly attenuated with distance and the presence of obstacles. To ensure drone detection from significant distances, the detection system should detect weak Wi-Fi signals. An RF amplification chain is then required. The implemented RF architecture is tailored to the system’s specifications. Indeed, the RDID uses a 10 MHz Wi-Fi channel fixed at 2437 MHz, while the DJI Drone ID uses a 5 MHz bandwidth frequency channel, which is on an unknown hopping channel in the 2.4 GHz or 5.8 GHz frequency bands. The three amplification stages account for these frequencies—the 2437 MHz channel, the full 2.4 GHz, and 5.8 GHz bands. The 2.4 GHz stage is divided into two parts by a two-way RF splitter, whereas the 5.8 GHz stage is transmitted without splitting, as illustrated in Figure 5. A Wi-Fi channel 6 cavity filter is included to capture only RDID packets. In addition, the remaining architecture is used to receive other communication packets, including the DJI Drone ID. Low-noise amplifiers (LNAs) are employed to amplify the RF signals received by the antennas, maximizing the signal-to-noise ratio (SNR). Following amplification, the signal is routed to a filter that eliminates non-useful frequencies according to the channels, making it ready for processing.

## 6. Detection and Tracking Software System

In this section, we provide a detailed description of the software tools used for the detection and identification of drone Wi-Fi signals. This section focuses on explaining the different software components and their functionalities in the context of drone detection and tracking.

### 6.1. Wi-Fi Packet Capture

#### 6.1.1. Aircrack-ng

Aircrack-ng is an open-source wireless network scanner used to detect and address vulnerabilities in wireless networks. It is a comprehensive suite of tools intended to assess the security of Wi-Fi networks, providing various features such as traffic monitoring and packet capture, and export of data for analysis. Aircrack-ng is specifically employed in this work to activate both interfaces (Panda/Intel Wi-Fi wireless chipsets) in monitor mode, allowing them to scan and monitor all the Wi-Fi channels.

#### 6.1.2. Wireshark

Wireshark is an open-source network analysis tool. It decodes captured frames and understands the different structures of communication protocols. In this work, Wireshark is employed to parse and decode the RDID packet at 2437 MHz.

#### 6.1.3. Kismet

Kismet is an open-source wireless network and sniffer that identifies and associates access points with wireless clients without emitting detectable frames. It employs a radio channel hopping algorithm to determine the maximum number of available networks. The hopping sequence, which can be customized, enables capturing more packets. In this work, Kismet supports the parsing and decoding of enhanced Wi-Fi DJI Drone ID frames due to its frequency hopping modulation capabilities.

### 6.2. Method for Identifying Wi-Fi Drone Nodes

Another utility of the “airckrack-ng” tool is the use of the “airodump-ng” program to capture raw IEEE Wi-Fi 802.11 frames. It is particularly suitable for collecting initialization vectors or handshakes. Every node has its own extended service set identifier (ESSID) and basic service set identifier (BSSID). The ESSID stands for the identifying name of an 802.11 wireless network, and the BSSID stands for basic service set identifier, and it is the media access control (MAC) physical address of a node. So, it is possible to distinguish drone signals from other node signals using either ESSID or BSSID.

**Figure 6:**
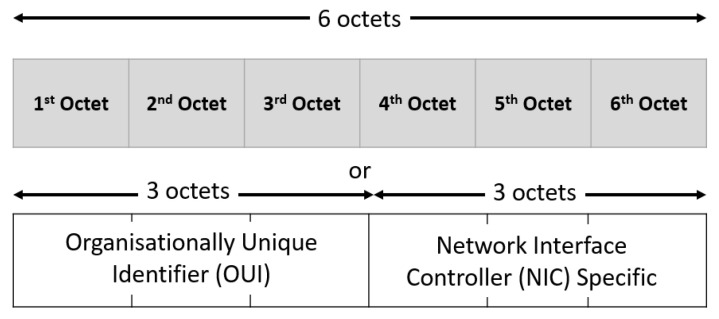
MAC address format [55].

As illustrated in Figure 6, the MAC address is made up of 6 bytes, or 48 bits. Each network card manufacturer is assigned a unique 3-byte identifier code, called the organizationally unique identifier (OUI). The manufacturer then assigns a unique value for the last 3 bytes to ensure that each MAC address is one-of-a-kind worldwide. Therefore, the first three bytes of the MAC address serves as an identifying characteristic of the supplier, which allows us to detect the drone’s presence. To sum up, this technique requires a Wi-Fi drone MAC address database [54] for detection. This method offers several benefits. Firstly, it is independent of the size and the drone material. Secondly, it does not require a LoS between the drone and the sensor. However, this means maintaining an up-to-date database of all MAC addresses for new UAVs.

### 6.3. Decoding Telemetry Packets

This section describes the method for decoding Wi-Fi RDID and enhanced Wi-Fi DJI Drone ID packets. To begin with, we put the Panda and the Intel wireless cards into monitor mode. This is achieved using the “aircrack-ng” tool with the following Linux command: “sudo airmon-ng start $<name_wireless_card>”. Once in monitor mode, the cards capture all traffic. To focus on analyzing specific packets, a series of filters is applied.

#### 6.3.1. Decoding Wi-Fi RDID Packet

**Figure 7:**
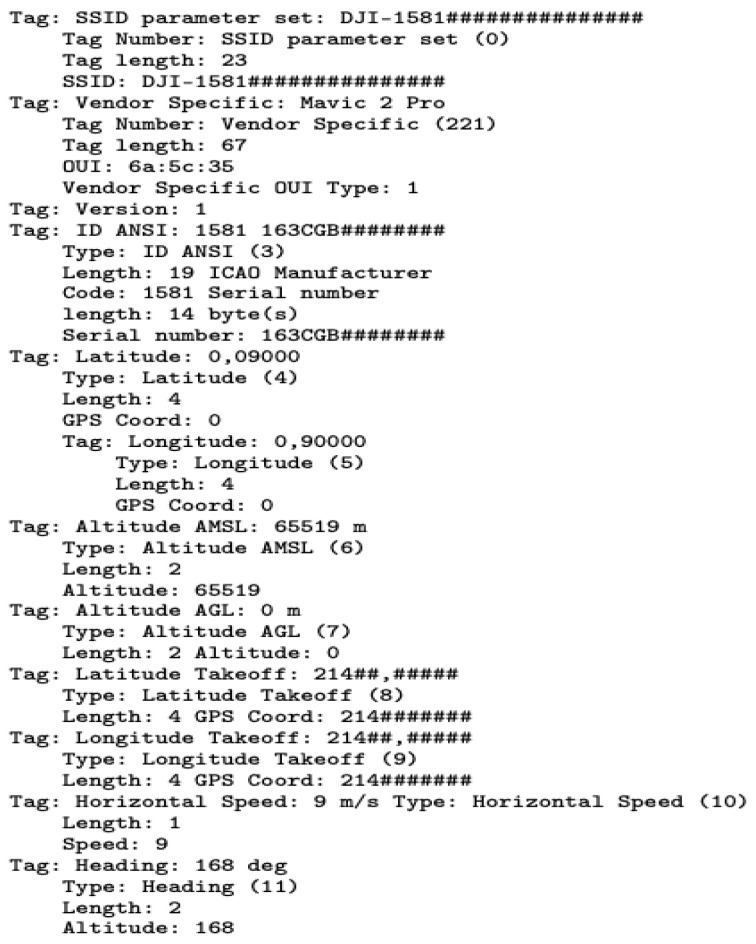
The Output PCAP file from decoding the Mavic 2 Pro RDID packet. Sensitive information was partly replaced by ’#’ symbol, such as location, MAC address, and serial number.

The RDID is broadcast unencrypted via the IEEE 802.11 wireless management flag as a beacon frame with an OUI of “6a:5c:35”. To isolate Wi-Fi beacon frames from the captured traffic, the command “wlan.fc.type_subtype == 0x08” is used. In addition, filtering is achieved with the BSSID command “wlan.bssid[0:3] == 60:60:1F”. To confirm the presence of the RDID, we check whether the OUI tag “6a:5c:35” is present in the decoded packet [56]. For example, Figure 7 displays the Mavic 2 Pro RDID packet capture (PCAP) file after applying these Wireshark commands.

The PCAP contains information about the drone’s location, altitude, speed, heading, and other related parameters. The packet has been decoded and contains various tags that describe the different parameters. The Radiotap header provides information about the captured packet, such as the interface, ID, and length. The IEEE 802.11 beacon frame indicates that this packet contains wireless management information. The fixed parameters include the timestamp, beacon interval, and capabilities information. The tagged parameters provide additional details about the drone, such as the service set identifier (SSID)(DJI-1581###############), latitude, longitude, altitude above mean sea level (AMSL), altitude above ground level (AGL), latitude takeoff, longitude takeoff, horizontal speed, and heading. Furthermore, there are vendor-specific tags that provide information about the drone manufacturer and serial number.

#### 6.3.2. Decoding Enhanced Wi-Fi DJI Drone ID Packet

DJI Wi-Fi drones include a standard packet addition known as a DJI Drone ID, which is broadcast through an OUI tag of “26:37:12” in the IEEE 802.11 beacon [57]. Two packet types are used; packets with a subcommand of 0 × 10 include flight telemetry and location, while packets with a subcommand of 0 × 11 include user-entered information about the drone and the flight [42]. These packets are alternately sent down the Wi-Fi link every 200 ms. Thanks to the OUI tag and the knowledge of the subcommands, Kismet can detect a DJI Drone ID packet data and classify it as “uav.device”. Kismet allows tracking using a list of drone MAC addresses by retrieving a UAV’s telemetry history. For more details, the DJI enhanced Wi-Fi Drone ID packet structure with the decoding process is explicitly detailed in references [41,42].

**Figure 8:**
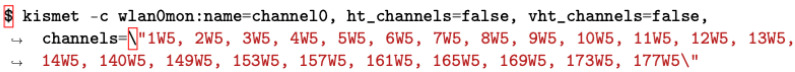
Monitor FHSS 5 MHz Wi-Fi signals using Kismet on the 2.4 GHz and 5.8 GHz bands.

Knowing that a DJI enhanced Wi-Fi Drone ID occupies a 5 MHz bandwidth, Wi-Fi channels can be scanned with Kismet from 1 to 177 in the 2.4 GHz and 5.8 GHz frequency bands using the following bash command [41] shown in Figure 8.

**Figure 9:**
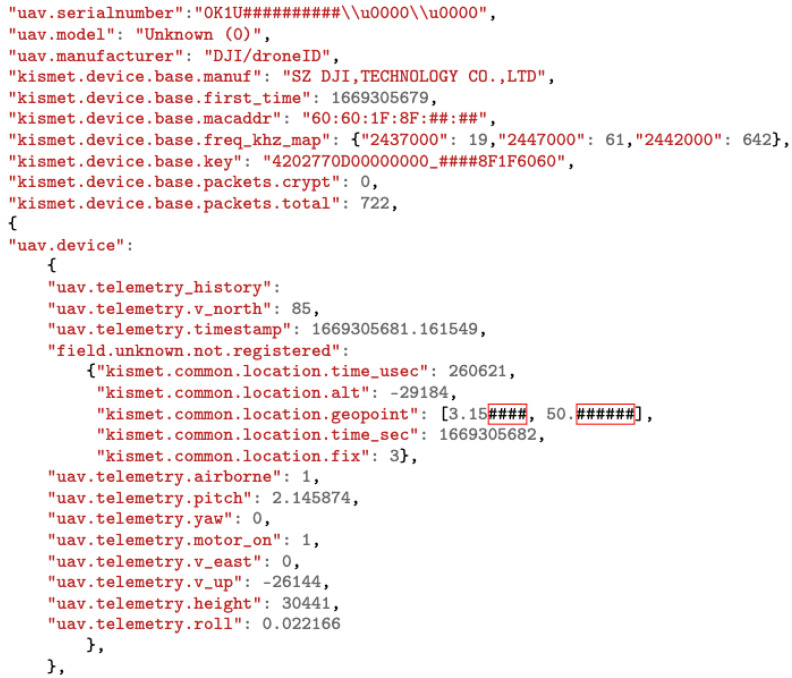
The Output JSON file from decoding DJI enhanced Wi-Fi Drone ID. Sensitive information was partly replaced by ’#’ symbol, such as location, MAC address, and serial number.

By using the Kismet_Rest application programming interface (API) [58] on Python, it is possible to retrieve decoded DJI Drone ID packet data from the Kismet server in the form of a JavaScript object notation (JSON) file as shown in Figure 9.

This packet contains information about a DJI drone, including its serial number, model, and frequency histogram usage. Additionally, telemetry data are included such as the drone’s pitch, yaw, and roll, as well as its speed and altitude. It also includes a timestamp indicating when the telemetry data were recorded. This information can be used to track and analyze drone movements and behavior.

**Figure 10:**
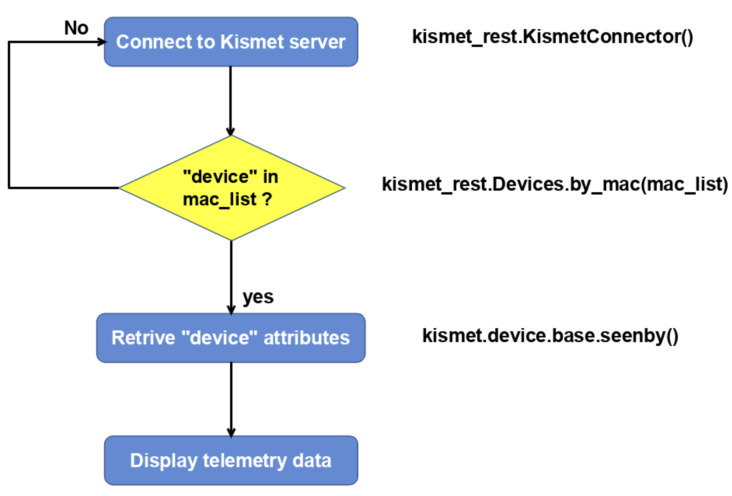
Flowchart of the Kismet REST Python code.

The Python code connects to a Kismet server using the kismet_rest API to retrieve information about a wireless detected device with a specific MAC address. As shown in the flowchart (Figure 10), the code performs several steps. First, the code imports the required kismet_rest module. Then, it creates a KismetConnector instance and establishes a connection to the Kismet server. Next, the code defines a list of MAC address masks, stored in the mac_list variable, for the devices that need to be detected. If a device is detected, the code retrieves information such as the device name, full MAC address, and manufacturer using the kismet_rest.Devices.by_mac function and providing the MAC list. Additionally, the code employs the kismet.device.base.seenby function to access multiple dictionary attributes.

## 7. Experimental Setup

**Figure 11:**
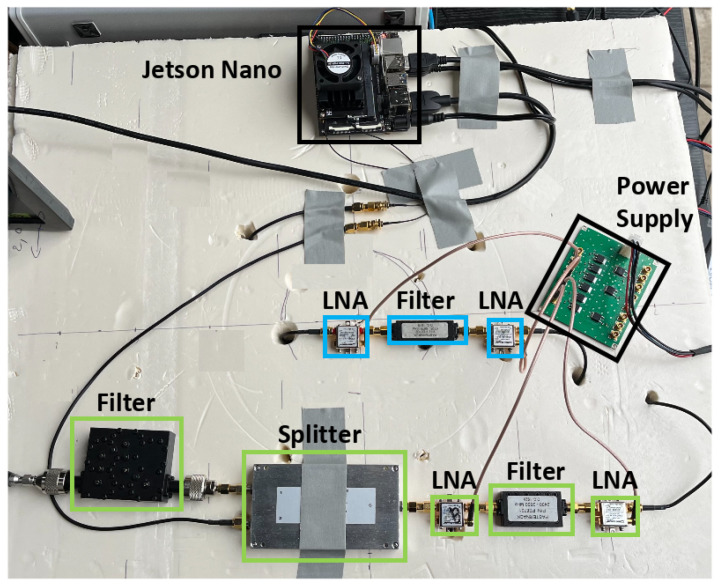
Illustration of the experimental setup.

In this section, we present the experimental setup employed to examine the detection and tracking capabilities of drones. As illustrated in Figure 11, the system incorporates both active and passive components within its RF chains. The specifications of the setup RF components are detailed in Table 2. The receiver chain is linked to the Jetson device for processing.

**Figure 12:**
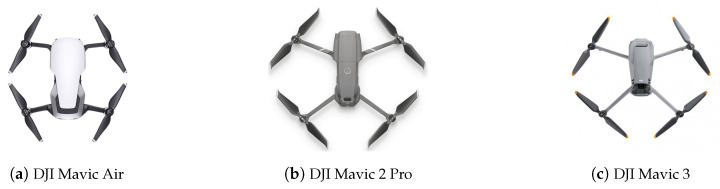
Drone models used in the experiments.

Figure 12 illustrates the drones tested in the experiments. These drones are the DJI Mavic Air, which is a Wi-Fi drone equipped with the enhanced Wi-Fi DJI Drone ID, and the DJI Mavic 2 Pro and DJI Mavic 3, which are OcuSync drones equipped with an RDID. Each drone selected employs a specific RF protocol and then represents a different category of drone. Their specific characteristics are outlined in Table 3. The drones chosen for the study are representative of many popular drones on the market due to their use of the recent RF protocols (Wi-Fi, enhanced Wi-Fi, and OcuSync). By focusing on these RF protocols, rather than on several specific drone models, our results can be extrapolated to a wide variety of drone models that share the same communication standards.

## 8. Detection and Tracking Assessment

Our objective is to evaluate the performance of the drone detection and tracking system across long distances. We aim to determine the maximum detection and tracking ranges for various drone models. These measurements provide insights into the system’s capabilities and limitations. These tests were conducted under different weather conditions and at various times of the day.

### 8.1. Methodology for Conducting the Measurement Campaign

**Figure 13:**
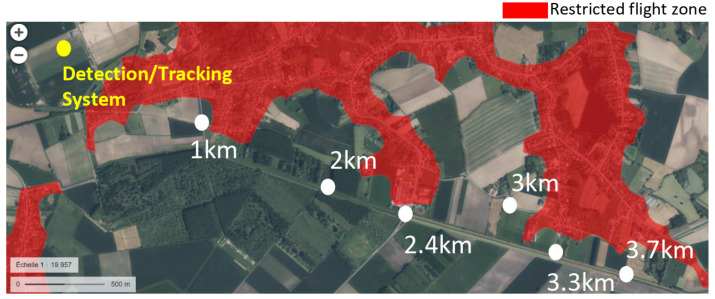
Outdoor mapping for long-distance drone experiments: system location (yellow spot) and pilot/drone positions (white spots).

This section describes the methodology for measuring distances of detecting and tracking the UAVs. An outdoor test site where drone flights are permitted was selected. It is located in a village in the north of France. There were a few sources of radio frequencies in the vicinity, such as cellular network base stations and Wi-Fi access points located in homes close to the test site. The detection system was approximately 1 km away from a small forest. This forest, composed of trees ranging from 20 to 30 m in height, was located in the intended flight path of the drones. Despite the proximity of these potential sources of interference, the site was deemed suitable for accommodating various flight scenarios. We also obtained authorization to fly drones in the unrestricted airspace shown in Figure 13.

**Figure 14:**
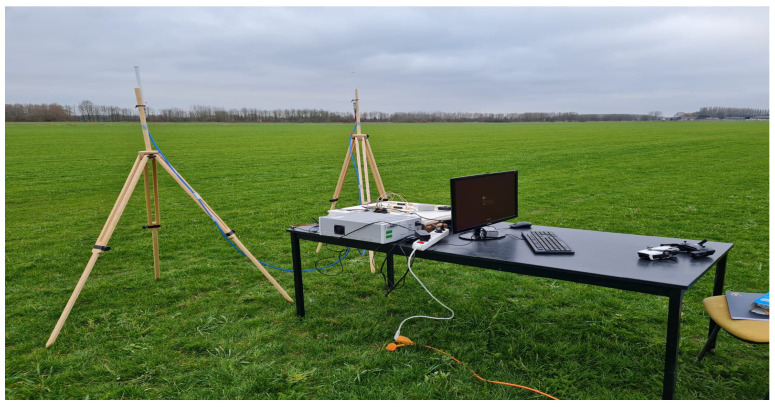
The outdoor test bed perspective.

We positioned the reference point at the location of the detection system, which is denoted by a yellow spot in Figure 13. The detection system is equipped with two omnidirectional receiving antennas and placed in an open-field environment. These antennas were mounted on 2.6 m tripods. The test bed perspective is shown in Figure 14.

Each time, the drone and the RC were positioned at different locations, indicated by white spots on the map in Figure 13. For some drones, we added between the white spots other positions for more measurements.

### 8.2. Performance of the Drone Detection and Tracking System

We gradually increased the drone’s altitude from the ground at each position. At the same time, we monitored the system’s ability to detect and retrieve telemetry information. This systematic approach allowed us to determine the minimum and maximum altitudes required for successful drone detection and tracking, depending on the distance from the reference point. In this section, we study the drone’s trajectory and measure the distances between the detected drones and the system. To assess the system’s accuracy, we calculate the errors between the actual and estimated trajectories, as well as the relative errors for the estimated altitude and speed of each drone. Additionally, for every identified drone, we calculated the remaining time to react to secure a designated area, given a specified protection radius.

#### 8.2.1. Measuring Detection Maximum Range and Altitude Interval

**Figure 15:**
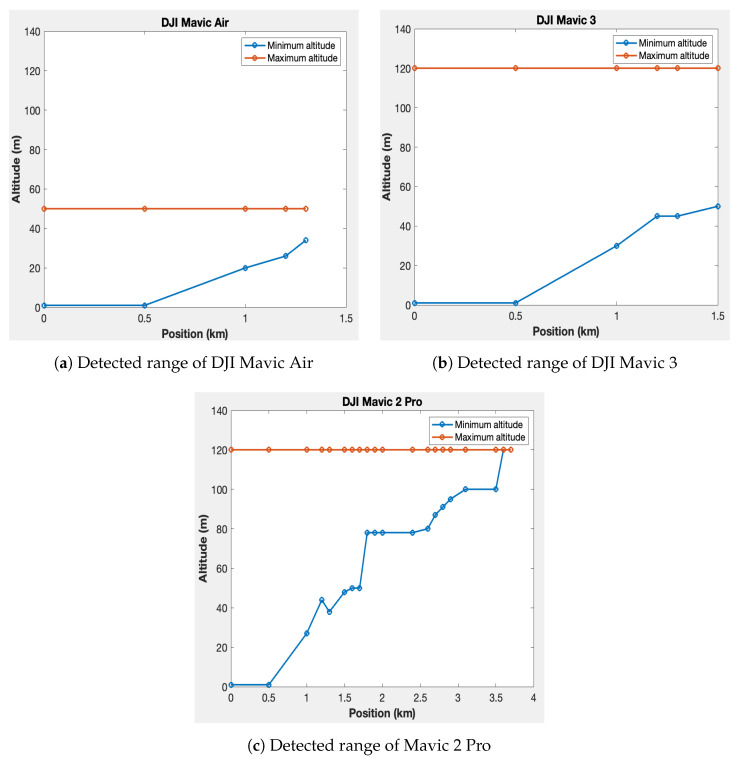
Measured detection ranges and interval altitudes of each drone.

Figure 15 presents the results for the Mavic Air, Mavic 3, and Mavic 2 Pro drones, respectively. In each figure, the blue curve represents the minimum altitude for detection at different distances from the reference, while the red curve represents the maximum altitude. The maximum altitude curve remains flat because the drones have altitude restrictions. The Mavic Air is limited to a maximum altitude of 50 m, while the Mavic 3 and Mavic 2 Pro have a maximum altitude of 120 m. The maximum detection distances achieved are 1.3 km for the Mavic Air, 1.5 km for the Mavic 3, and 3.7 km for the Mavic 2 Pro.

#### 8.2.2. Estimating Drone Positions

**Figure 16:**
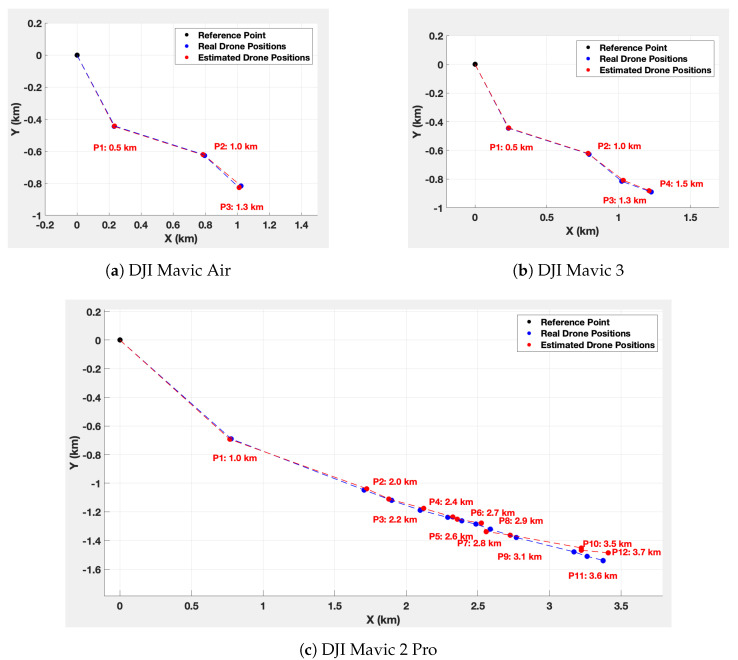
Precise drone positions, estimated drone positions, and the Haversine distances from the system estimation.

To estimate the drone positions, an automated code distinguishes the telemetry packets for each detected drone thanks to the drone identifier. Then, for each drone, the telemetry packets are sorted by time of reception to trace the trajectory. The latitude and longitude (φ, λ) information are extracted and converted into 2D Cartesian coordinates (*x*, *y*) using Equation (1). Figure 16 illustrates the real positions and the estimated positions for each drone.

(1) $$\left\{\begin{array}{c} x=R_{\mathrm{earth}}\cos\left(\varphi \right)\cos\left(\lambda \right)\\ y=R_{\mathrm{earth}}\cos\left(\varphi \right)\sin\left(\lambda \right) \end{array}\right.$$

To perform comparisons between the estimated and the real positions, we calculate the Euclidean distance error. This metric measures the discrepancy between the real positions and the estimated positions of each drone. Denoted by e(P), the Euclidean distance error is obtained using the following formula:

(2) $$e\left(P\right)=\sqrt{{\left(x_{\mathrm{estimated}}\left(P\right)-x\left(P\right)\right)}^{2}+{\left(y_{\mathrm{estimated}}\left(P\right)-y\left(P\right)\right)}^{2}}$$

where $\left(x_{\mathrm{estimated}}\left(P\right),y_{\mathrm{estimated}}\left(P\right)\right)$ are the estimated coordinates, and (x(P),y(P)) are the precise coordinates of the position of the drone *P*.

**Figure 17:**
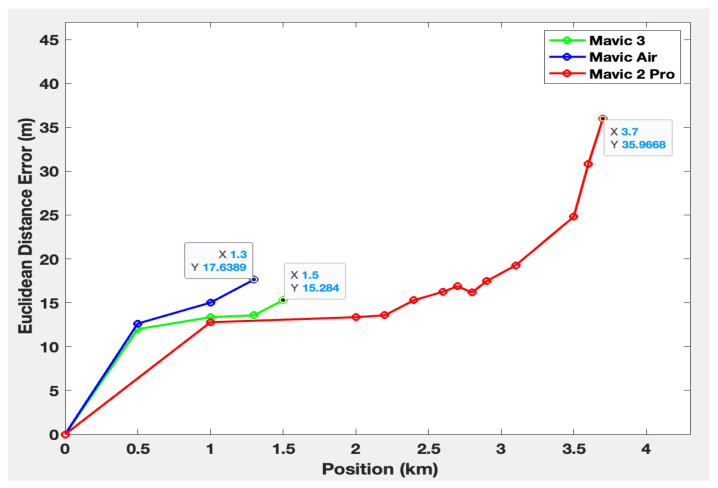
Euclidean distance error between real and estimated positions.

Figure 17 shows the Euclidean distance error between the estimated and the real positions.

#### 8.2.3. Estimation of Remaining Distance between the Drone and the System

To find out how far the drone is from the system, we use the Haversine equation (Equation (3)) [59]. The calculated distances are shown in Figure 16.

(3) $$\left\{\begin{array}{c} a=\sin^{2}\left(\frac{\Delta \varphi }{2}\right)+\cos\left(\varphi_{1}\right)\cos\left(\varphi_{2}\right)\sin^{2}\left(\frac{\Delta \lambda }{2}\right)\\ c=2\times \mathrm{atan}2\left(\sqrt{a},\sqrt{1-a}\right)\\ d=R_{earth}\times c \end{array}\right.$$

The Haversine equation calculates the distance *d* between two points on Earth, where $\varphi_{1}$ and $\varphi_{2}$ are the latitudes of the points, and Δφ and Δλ represent the differences in latitude and longitude, respectively, and $R_{\mathrm{earth}}$ represents the Earth’s radius. This formula considers the spherical Earth’s shape. Indeed, the traditional Euclidean distance calculations, being based on flat Cartesian coordinates, are not accurate for long distances. By considering Earth’s curvature, the Haversine formula provides more accurate distance measurements.

#### 8.2.4. Relative Error for Altitude and Speed Estimation

Let *X* represent the telemetry measured parameter whose relative error we want to evaluate. The formula for calculating the relative error can be written as follows:

(4) $$\xi_{X}\left(P\right)=100\times \left|\frac{X_{\mathrm{decoded}}\left(P\right)-X_{\mathrm{precise}}\left(P\right)}{X_{\mathrm{precise}}\left(P\right)}\right|$$

Equation (4) represents the relative error $\xi_{X}\left(P\right)$ for parameter *X* at position *P*. Here, $X_{\mathrm{decoded}}\left(P\right)$ denotes the decoded value of the parameter *X* at position *P*, and $X_{\mathrm{precise}}\left(P\right)$ signifies the reference parameter *X* at position *P*. As the pilot was in proximity to the drone at each position, we assume that the parameters received by the pilot and recorded in the phone’s telemetry history of the pilot represent accurate information about the flight. Thus, $X_{\mathrm{precise}}\left(P\right)$ is extracted from the telemetry information in the phone’s flight history. On the other hand, the measured parameters represent the decoded values provided by the detection system $X_{\mathrm{decoded}}\left(P\right)$.

To assess the errors in altitude and speed, we substitute *X* with the altitude H and the speed V of the tracked drone, estimated by the system. The formula becomes Equation (5):

(5) $$\left\{\begin{array}{c} \xi_{\mathrm{altitude}}\left(P\right)=100\times \left|\frac{H_{\mathrm{decoded}}\left(P\right)-H_{\mathrm{precise}}\left(P\right)}{H_{\mathrm{precise}}\left(P\right)}\right|,\\ \xi_{\mathrm{speed}}\left(P\right)=100\times \left|\frac{V_{\mathrm{decoded}}\left(P\right)-V_{\mathrm{precise}}\left(P\right)}{V_{\mathrm{precise}}}\right|. \end{array}\right.$$

**Figure 18:**
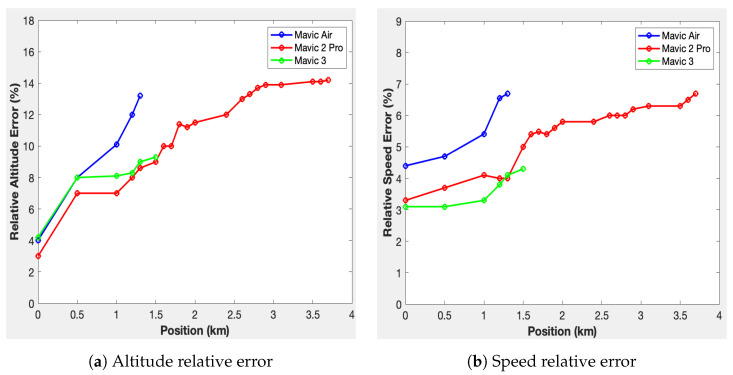
Relative error calculations.

The relative altitude and speed error curves obtained from this formula, expressed as a percentage for each position, are shown in Figure 18. The blue curve corresponds to the Mavic Air drone, the red curve represents the Mavic 2 Pro drone, and the green curve represents the Mavic 3 drone.

#### 8.2.5. Remaining Time to React

Thanks to the speeds of each drone from Table 3 and the maximum detection distance from Figure 15, we can compute the remaining time to neutralize a potentially dangerous drone, based on the system’s location in the protected site.

For instance, to protect a site with a 200 m radius, the computed reaction times would be 1.83 min for the Mavic Air, 1.03 min for the Mavic 3, and 2.92 min for the Mavic 2 Pro. This computation is performed using the following formula:

(6) $$T_{\mathrm{to}-\mathrm{react}}=\frac{d_{\mathrm{drone}}-r_{\mathrm{site}}}{v_{\mathrm{drone}}}$$

where $T_{\mathrm{to}-\mathrm{react}}$ represents the remaining time available to react, $d_{\mathrm{drone}}$ is the distance from the drone to the vulnerable site, $r_{\mathrm{site}}$ is the fixed radius of the site to protect, and $v_{\mathrm{drone}}$ is the maximum linear speed of the drone. This equation provides valuable insights into the time required to take appropriate actions based on the drone’s position and maximum linear speed. It offers a simplified representation of the remaining reaction time once the drone is detected.

Equation (6) considers a worst-case scenario in which the drone travels at its maximum speed and approaches the vulnerable site along a direct linear trajectory.

## 9. Discussion about the Results

The maximum detection ranges for the Mavic Air, Mavic 3, and Mavic 2 Pro are, respectively, 1.3 km, 1.5 km, and 3.7 km. These results highlight the system’s enhanced ability to detect the Mavic 2 Pro compared to the others. This can be explained by the fact that signal capture also depends on the signal transmission power specific to each drone. Moreover, the minimum detection altitude of the three drones increases as they move away from the system. This might be attributed to the system’s antennas, whose radiation patterns are slightly oriented upwards. In addition, the position estimation error tends to increase as the drones move further away from the detection system. The maximum error is 17 m at 1.3 km for the Mavic Air, 15 m at 1.5 km for the Mavic 3, and 35 m at 3.7 km for the Mavic 2 Pro. The higher error for the Mavic 2 Pro is, therefore, probably due to its greater detection distance. Nevertheless, among the configurations tested, the speed and position estimation errors were no greater than 7% and 14%.

**Figure 19:**
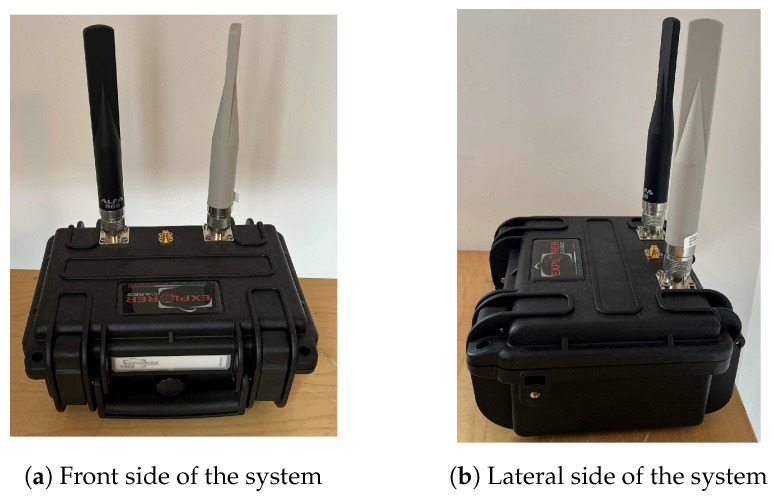
Front and lateral sides of the system.

Moreover, the Haversine equation can be used to estimate the remaining distance between the detected UAV and the detection system, using the longitude and latitude data decoded from the frames. Considering the protection of a 200 m radius area with the detection system at its center, the estimated reaction times are 1.83 min for the Mavic Air at a distance of 1.3 km, 1.03 min for the Mavic 3 at 1.5 km, and 2.92 min for the Mavic 2 Pro at 3.7 km. These values, therefore, make it possible to specify the expected performance of the drone interception solution to be implemented. Finally, the detection and tracking system, depicted in Figure 19, can be integrated into a single module offering portability, ease of deployment, and adaptability, making it a relevant tool for organizations and agencies monitoring drone activity in their airspace.

## 10. Conclusions and Future Work

In conclusion, the conducted study provides insight into drone detection and tracking systems, focusing particularly on the Mavic Air, Mavic 3, and Mavic 2 Pro drones. The research outlines the detection range for these drones, with maximum detected distances at 1.3 km, 1.5 km, and 3.7 km, respectively. The detection capabilities are influenced by factors such as the drone’s transmission power and multipath propagation, contributing to the variation in the observed results. The research notes an increase in position estimation error as drones move further away from the system. The relative error in estimating speed and altitude also increased with distance, though these did not exceed 7% and 14%, respectively. The use of the Haversine equation in estimating the remaining distance between the detected drone and the system yielded promising results. The system was also tested in a hypothetical scenario involving securing an area with a 200 m radius. The remaining reaction times for different drones were computed, providing useful data for applications aiming at the interception of unauthorized drones.

This research presents a system that integrates detection, classification, and localization functionalities in a single module. This study demonstrated the system’s capability to differentiate drone signals and track their movements. While the study showcased the system’s potential in managing drone activities and its potential contribution to public safety and security, some areas need improvement.

To enhance our solution, we plan future expansions. These include integrating real-time decoding of OcuSync DJI Drone IDs for other DJI drone identification and classification, incorporating AI models for detecting other drones that are not equipped with Drone IDs, improving the RF receiver to extend system coverage, supporting different frequency bands to utilize the system indifferent geographic region, and the possibility to connect the system with a jamming device [60,61] for drone interception and man–machine interface (MMI). These planned expansions hold promising prospects for enhancing safety and responsiveness in drone detection and mitigation.

## Figures and Tables

**Table 1:** Overview of technologies for drone detection and tracking [17,24].

Type	Definition	Pros	Cons	References
EO/IR Imaging	Visual-based detection using cameras to capture and analyze drone presence, covering the visible and IR spectrum from 3 MHz to 300 GHz.	Use of computer-vision AI algorithms; Availability of high-resolution cameras; Real-time tracking.	Limited range; Weather-dependent; LoS is required; Low-light issues.	[25,26]
Acoustic	Auditory detection, leveraging microphone arrays to discern drone-produced sounds, covering the spectrum from 20 Hz to 20 kHz.	Passive detection; LoS is not required; Low power consumption.	Sensitive to ambient noise; Extremely short detection range; Dependency on drone noise.	[27,28]
RaDaR	RCS reflection or micro-doppler signature-based detection, with a bandwidth used from 3 MHz to 300 GHz.	Long detection range; 360-degree coverage; All-weather operation.	Large RCS; Confusion with other flying objects, such as birds; LoS is required; Expensive.	[18,29,30]
Radio Frequency	Monitoring the radio frequency spectrum, identifying drone-specific communication signals.	Passive detection; LoS is not required; Low complexity and easy to implement; Easier to upgrade due to modular implementation; Possibility of decoding communication signals; Potential to localize the pilot.	Requires a large database of RF drone/RC signals; Confusion with other RF communications, especially in complex environments; Vulnerable to illegally modified RF hardware drones that exceed receiver capabilities.	[31,32,33]

**Table 2:** RF component specifications.

Component	Frequency Range	Specifications
Dual-Band Antennas	2.4 GHz/5 GHz	Omnidirectional;Gain at 2.4 GHz: 5 dBi; Gain at 5 GHz: 9 dBi
Low-Noise Amplifier	0.5–8 GHz	Low noise: 1.4 dB @ 2 GHz; High IP3: +34 dBm; Gain flatness: ±0.9 dB over 0.5 to 7 GHz @6V
Cavity Bandpass Filter	2.4 GHz	Center frequency: 2437 MHz; Bandwidth: 25 MHz; Maximum insertion loss: 1.5 dBm
Bandpass Filter	2.4–2.5 GHz	Bandwidth: 400 MHz; Insertion Loss: 1 dB; Power capacity: 5 W; Voltage standing wave ratio (VSWR): 1.50:1
Bandpass Filter	5.725–5.875 GHz	Bandwidth: 150 MHz; Maximum insertion loss: 1 dB; Maximum power: 5 W
Power Splitter/Combiner	350–6000 MHz	Power capacity (as splitter): 25 W; Insertion loss: 0.9 dB

**Table 3:** Drone Specifications.

Drone	OUI ID	RF Protocol	Linear Maximum Velocity	RDID	DJI Drone ID
DJI Mavic Air	60:60:1F	Enhanced Wi-Fi	10 m/s	×	✓(Enhanced Wi-Fi)
DJI Mavic 2 Pro	60:60:1F	OcuSync 2.0	20 m/s	✓(Wi-Fi)	×
DJI Mavic 3	60:60:1F	OcuSync 3.0+	21 m/s	✓(Wi-Fi)	✓(OcuSync)

## Data Availability

https://gist.github.com/aallan/b4bb86db86079509e6159810ae9bd3e4, accessed on 31 August 2023.

## Abbreviations

The following abbreviations are used in this context:

UAV	Unmanned aerial vehicle
GPS	Global Positioning System
LiDAR	Light detection and ranging
RaDaR	Radio detection and ranging
C-UAS	Counter-unmanned aerial system
LoS	Line of sight
GA-XGBoost	Genetic algorithm-extreme gradient boosting
IMU	Inertial measurement unit
GoF	Goodness-of-fit
DRNN	Deep recurrent neural network
YOLO	You only look once
CNN	Convolutional neural network
SVM	Support vector machine
AI	Artificial intelligence
SDR	Software-defined radio
RDID	Remote drone identification
UAS	Unmanned aerial system
RC	Remote controller
ISM	Industrial, scientific, and medical
OFDM	Orthogonal frequency division multiplexing
FHSS	Frequency hopping spread spectrum
ASTM	American Society for Testing and Materials
ASD-STAN	Aerospace and Defence Industries Association of Europe
FAA	Federal Aviation Administration
FRIAs	FAA-recognized identification areas
GNSSs	Global navigation satellite systems
LNAs	Low-noise amplifiers
SNR	Signal-to-noise ratio
ESSID	Extended service set identifier
BSSID	Basic service set identifier
MAC	Media access control
OUI	Organizationally unique identifier
PCAP	Packet capture
SSID	Service set identifier
AMSL	Above mean sea level
AGL	Above ground level
API	Application programming interface
JSON	JavaScript object notation
MMI	Man–machine interface
ANRT	Association Nationale de la Recherche et de la Technologie
RCS	Radar cross-section
IR	Infrared
EO	Electro-optical
VSWR	Voltage standing wave ratio
